# Risk factors for chronic post-traumatic stress disorder development one year after vaginal delivery: a prospective, observational study

**DOI:** 10.1038/s41598-017-09314-x

**Published:** 2017-08-18

**Authors:** Loïc Sentilhes, Françoise Maillard, Stéphanie Brun, Hugo Madar, Benjamin Merlot, François Goffinet, Catherine Deneux-Tharaux

**Affiliations:** 1 Department of Obstetrics and Gynecology, Bordeaux University Hospital, Bordeaux, France; 2 Inserm UMR 1153, Obstetrical, Perinatal and Pediatric Epidemiology Research Team (EPOPé), Center for Epidemiology and Statistics Sorbonne Paris Cité (CRESS), DHU Risks in pregnancy, Paris Descartes University, Paris, France; 3 Port-Royal Maternity Unit, Department of Obstetrics and Gynecology, Cochin University Hospital, APHP, Paris, France

## Abstract

Our study aimed to assess the prevalence of post-traumatic stress disorder (PTSD) after childbirth one year after vaginal delivery and to identify characteristics of women and deliveries associated with it. Questionnaires were mailed a year after delivery to 1103 women with prospectively collected delivery and postpartum data, including a question on day 2 assessing their experience of childbirth. PTSD was assessed a year later by the Impact of Event and Traumatic Event Scales; 22 women (4.2%, 95%CI 2.7–6.3%) met the PTSD diagnostic criteria and 30 (5.7%; 95%CI 3.9–8.0%) PTSD profile criteria. Factors associated with higher risk of PTSD profile were previous abortion (aOR 3.6, 95%CI 1.4–9.3), previous postpartum hemorrhage (Aor 5.3, 95%CI 1.3–21.4), and postpartum hemoglobin <9 g/dl (aOR 2.7, 95%CI 1.0–7.5). Among 56 women (10.3%) reporting bad childbirth memories at day 2 postpartum, 11 (21.1%) met PTSD diagnosis and 11 (21.1%) PTSD profile criteria a year later, compared with 11 (2.4%) (*P* < 0.001) and 18 (3.8%) (*P* < 0.001), respectively, of the 489 (87.7%) women with good memories. PTSD is not rare at one year after vaginal delivery in a low-risk population. A simple question at day 2 post partum may identify women most at risk of PTSD and help determine if early intervention is needed.

## Introduction

Psychiatric symptoms after delivery are classically divided into three categories: postpartum blues, postpartum depression, and postpartum psychosis. Another psychiatric issue that has drawn increasing attention over the last decade is post-traumatic stress disorder (PTSD), an anxiety disorder^1^, after childbirth.

PTSD is a common sequela of traumatic events such as physical or sexual assault^1^. Although childbirth is considered a positive experience, there is increasing evidence that childbirth may be a cause of PTSD^2^, with some women reporting intense fear, helplessness, and threat to their integrity during labor. Of note, in most cases it is difficult to affirm that the PTSD observed after childbirth is caused by the delivery itself, as it might be the consequences of a pre-existing/lifetime PTSD, caused by another incident trauma − unknown, insufficiently assessed, or hidden by the woman herself − that becomes symptomatic following childbirth. Knowledge about the prevalence of PTSD symptoms following childbirth – whether related to or associated with it– nonetheless remains limited. The condition has been estimated to range from 2–6% at about 6 weeks postpartum and 1.5% at 6 months after birth^3^. The prevalence at one year is unknown^4^. PTSD following childbirth is highly co-morbid with depression^5^ and impacts the couple relationship^6^. Postnatal psychiatric disorders may also be an important public health issue^4^ because of the long-term negative impact of such maternal mental health problems on child development^7–9^, including impairment of the mother–infant relationship^10^, delayed intellectual development^11, 12^, and psychiatric disorders in children^13^. This situation highlights the need to identify women at risk for subsequent PTSD during the early postpartum period.

The wide range of individual, medical, and social factors that can interact to affect women’s experiences of childbirth can make this experience complex. Previous research into possible predictors or risk factors for postpartum PTSD has found it to be associated with some characteristics of the woman or her pregnancy (pre-existing psychiatric condition, prior traumatic experiences, anxiety trait, nulliparity, or unplanned pregnancy) and the labor and delivery (preterm delivery, physical pain, emergency cesarean, instrumental delivery, high level of fear for self and/or the baby, loss of control, or low support from partner or staff)^4, 14^. Nevertheless, the overall quality of these studies is moderate to low^4^. Most were retrospective and used postnatal recruitment, both factors that necessitate caution about the quality of the prenatal and intrapartum data reported^4^. On the whole, little is known regarding the impact of obstetric interventions (induction and duration of labor, epidural, episiotomy, perineal tear, etc.) on psychological outcomes, except for emergency situations.

The first aim of our study was to determine the prevalence of PTSD one year after vaginal delivery in a low-risk sample from a large prospective cohort. The secondary aim was to identify characteristics of labor and delivery as well as women’s early postnatal experience of childbirth associated with a PTSD profile at one year postpartum.

## Methods

### Participants

This prospective study included all women who were enrolled in the TRACOR trial^15^ and gave birth at the Angers University Hospital, in Angers, France. TRACOR was a randomized controlled trial (RCT) conducted in five hospitals between January 1, 2010, and January 31, 2011; it found that controlled cord traction in the third stage of labor did not affect the incidence of postpartum hemorrhage in low-risk women (who were aged 18 or older with a singleton fetus, gave birth at 35 or more weeks’ gestation, and understood French well) after a vaginal delivery. Women with severe hemostatic disorders, placenta previa, in utero fetal death, or multiple gestations were excluded. In this trial, women’s experience of childbirth was assessed by a self-administered questionnaire on day 2 postpartum^15^.

### Interventions and data collection

One year after delivery, all women (n = 1103) were mailed a self-administered questionnaire that included the Traumatic Event Scale (TES)^16–19^, Impact of Event Scale (IES)^20^, Spielberger State-Trait Anxiety Inventory, form Y (STAI)^21^, and Edinburgh Postnatal Depression Scale (EPDS)^22^ (see below). After two months, the questionnaire was mailed again to non-respondents. The Angers Hospital Faculty Review Board and Ethics Committee approved the study (number 2011/10), which was conducted in accordance with relevant guidelines and regulations. All patients gave their informed consent for use of the data collected.

The characteristics of the women, labor, delivery, third stage of labor (with special attention to blood loss, measured by a collector bag), hemoglobin level at day 2 postpartum, and neonatal status were extracted from the TRACOR database^15^), together with the responses to the questionnaire self-administered on day 2 after delivery, in particular, the question “Today, what are your memories of your childbirth?” Women could answer this question on a five-point Likert-type scale (excellent, good, intermediate, bad, or very bad). For our analysis, bad memories of delivery were defined by a “bad” or “very bad” response. A research assistant, independent of the local medical team, collected these data; the quality of collected data was checked in each center for a 10% random selection of the women included and for all women with postpartum hemorrhage^15^.

### Traumatic Event Scale

PTSD was measured by the TES^16–19^, developed specifically for PTSD following childbirth and in accordance with the Diagnosis and Statistical Manual of Mental Disorders fourth edition (DSM-IV) criteria for PTSD. According to the DSM-IV^1^, the stressor for PTSD should be an event that involves actual or threatened death or serious injury or a threat to the physical integrity of oneself or others, and the person’s response must involve intense fear, helplessness, or horror (criterion A). The three categories of PTSD symptoms are persistent re-experiencing of the trauma (criterion B), persistent avoidance of stimuli associated with the trauma (criteria C), and symptoms of increased physiologic arousal (criterion D). Symptoms must last for at least one month (criterion E) and cause impairment in daily life (criterion F)^1^. The main advantage of TES is its inclusion of the stressor criterion (criterion A) and all symptom criteria for PTSD (criteria B, C, D, E, and F)^16–19^. The internal consistency of the scale was estimated by Cronbach’s α, calculated at 0.87. For criterion A, the delivery was specified as the event of interest and was assessed by four questions formulated as follows: (1) “the childbirth was a trying experience”; (2) “the childbirth was a threat to my physical integrity”; (3) “during the childbirth I was afraid that I was going to die”; (4) “during the childbirth, I felt anxious/helpless/horrified”^16, 17^. Four possible answers for each statement were proposed: “not at all”; “somewhat”; “moderately”; “very much so”. Criterion A was met by the responses “moderately” or “very much so” for items (1), (2) and/or (3) and (4)^16^. After criterion A, items assessed the 17 DSM-IV PTSD symptoms for criteria B, C, and D (i.e., intrusive thoughts, avoidance, and arousal, respectively). Women were asked to report the frequency of the symptoms described in each item by marking one of four possible answers: “never/not at all”; “rarely”; “sometimes”; “often”^16^. Respondents rated the 17 symptoms on a four-point scale (0–3); a minimum score of 2 (“sometimes”) was considered to reflect the presence of a symptom^16^. In line with DSM-IV, the criteria for PTSD symptoms were considered met when a respondent reported at least one of five re-experiencing symptoms (criterion B), three of seven avoidance symptoms (criterion C), and two of five hyperarousal symptoms (criterion D)^16–19^. If the symptoms were present for at least one month, criterion E was met; if the woman rated the degree to which her life was affected by the symptoms greater than 5 on a 10-point scale, criterion F was met. The duration criterion of one month (DSM-IV criterion E) was met because the follow-up assessment was at one year after the birth.

The term PTSD diagnosis is used when all symptom criteria of the DSM-IV (A, B, C, D, E, and F) are met^16–19^. The term PTSD profile is used when all symptom criteria (B, C, and D)^1^ are met, but some other criteria (A, E or F) are missing^4^.

### Impact of Event Scale

Post-traumatic stress symptoms were also measured by the IES^20^, which has been validated in French^23^. Seven items measure intrusion (criterion B) and eight measure avoidance (criterion C). The IES does not cover the hyperarousal symptoms that are part of the DSM-IV criteria for PTSD (criterion D). An IES score of 26 or higher indicates very serious post-traumatic stress^24^. Other authors consider that a score greater than 19 is clinically significant and a score greater than 30 a marker of the need for specific care^25, 26^.

### Spielberger State-Trait Anxiety Inventory

We also aimed to assess the incidence of anxiety and depression in a low-risk population at one year after a vaginal delivery. Therefore, anxiety was assessed by the 20-item STAI^25^. The questionnaire has been adapted and validated in French^27^. Items were rated on a four-point Likert scale ranging from 1 (not anxious) to 4 (highly anxious), with overall scores ranging from 20 to 80. Sum-scores (20 items) were dichotomized at the top 25th percentile: scores greater than 44 were considered high trait anxiety and scores less than 45 as low trait anxiety.

### Edinburgh Postnatal Depression Scale

Moreover, the EPDS was used to assess depressive symptoms post partum^22^. This 10-item scale was first developed for assessing postnatal depression^22^ and was then validated in childbearing women^28^. The French version of the scale was validated in 1995^29^. Each item is scored on a four-point scale from 0 to 3, with minimum and maximum overall scores ranging from 0 to 30. We used the standard cutoff value of 12 to indicate depression^22, 28, 29^.

### Outcomes

The primary outcome was PTSD diagnosis and profile one year after a vaginal delivery. The main secondary outcomes were other objective measures of psychological symptoms at that time, i.e., anxiety and depression, as defined above.

### Statistical analysis

We compared the characteristics of the women, labor, delivery, immediate postpartum period, and neonates between respondents and non-respondents. Prevalence of PTSD by the TES and IES scales and depression at one-year postpartum were estimated with binomial 95% confidence intervals (95% CIs). We investigated the factors associated with PTSD profiles and IES scores of the potential collinearity of some variables, we built two multivariate models for each outcome including either risk factors for excessive 26 or higher by univariate and multivariate logistic regression analysis. Variables included in the multivariate analysis were those associated with PTSD on univariate analysis. Because bleeding – long labor, instrumental delivery, and episiotomy, or postpartum Hb less than 9 g/dl. Odds ratios (ORs) and their 95% CIs were calculated. The proportion of missing values was 6% or less for all variables. Differences were considered significant at *P* < 0.05. Stata SE 12 was used for all analyses.

### Details of Ethics Approval

The study was approved by the Angers Hospital Faculty Review Board and Ethics Committee (number 2011/10) and all patients gave their informed consent for use of the data collected.

## Results

Questionnaires were returned by 549 of 1103 women (49.8% response rate). Non-respondents were significantly younger than non-respondents, smoked and had had a previous abortion or instrumental delivery more often (Table 1). They also reported bad memories of childbirth more often at day 2 post partum (Table 1).

**Table 1:** Characteristics of woman, labor, delivery, immediate postpartum period, and neonates among respondents and non-respondents.

	Non-respondents (n = 554) n/N (%)	Respondents (n = 549) n/N (%)	P
**Woman characteristics**			
Age (years)			<0.001
<25	147/554 (26.5)	60/549 (10.9)	
25–34	337/554 (60.8)	400/549 (72.9)	
≥35	70/554 (12.7)	89/549 (16.2)	
French nationality	548/554 (98.9)	542/549 (98.9)	0.99
**Tobacco use**			
Never	357/548 (65.1)	393/545 (72.1)	0.001
Never during pregnancy	65/548 (11.9)	74/545 (13.6)	
Yes during pregnancy	126/548 (23.0)	78/545 (14.3)	
Body mass index (kg/m^2^)			0.15
<18.5	30/554 (5.4)	33/548 (6.0)	
18.5–24.9	375/554 (67.7)	397/548 (72.5)	
25.0–29.9	99/554 (17.9)	85/548 (15.5)	
≥30	50/554 (9.0)	33/548 (6.0)	
Nulliparous	246/554 (44.4)	254/549 (46.5)	0.48
Previous abortion	78/554 (14.1)	53/549 (9.7)	0.025
Previous postpartum hemorrhage	18/554 (3.2)	15/549 (2.7)	0.62
Uterine scar	47/554 (8.5)	32/549 (5.8)	0.09
Complication during pregnancy*	110/554 (19.9)	100/549 (18.2)	0.49
Hospitalization >24 h	55/554 (9.9)	53/549 (9.7)	0.89
**Labor characteristics**			
Induction of labor	88/554 (15.9)	85/549 (15.5)	0.95
Duration of labor >6 h	178/554 (32.1)	190/549 (34.7)	0.36
Epidural analgesia	540/554 (97.5)	526/549 (96.0)	0.17
Oxytocin during labor**	450/554 (81.2)	434/549 (79.2)	0.40
Instrumental delivery	96/554 (17.3)	67/549 (12.2)	0.02
Episiotomy	228/554 (41.2)	218/549 (39.7)	0.62
3^rd^- or 4th-degree perineal lacerations	7/554 (1.3)	7/549 (1.3)	0.99
Manual removal of placenta	22/554 (4.0)	32/549 (5.8)	0.15
Blood loss ≥500 ml	56/554 (10.1)	60/549 (10.9)	0.66
Blood loss ≥1,000 ml	17/554 (3.1)	14/549 (2.5)	0.60
**Immediate postpartum period**			
Postpartum hemoglobin at D2 [mean (SD)]	10.8 (1.4) (n = 548)	11.0 (1.4) (n = 540)	
Hemoglobin level <9 g/dl at D2	49/548 (8.9)	51/540 (9.4)	0.77
Bad memories of delivery	81/554 (14.8)	56/549 (10.3)	0.025
**Neonatal outcome**			
Birth weight (g) (mean [SD])	3340 (441) (n = 554)	3383 (425) (n = 548)	0.10
5-min Apgar score <7	3/554 (0.5)	1/549 (0.2)	0.62
pH <7.00	0	0	—
Transfer to neonatal intensive care unit	23/554 (4.1)	15/549 (2.7)	0.20

*Complication during pregnancy including thrombocytopenia, gestational diabetes, bleeding, hypertension disorders, preterm labor. **First and second stage.

One year after delivery, 22 (4.2%; 95% CI 2.7–6.3%) women met the criteria for PTSD diagnosis and 30 (5.7%; 95% CI 3.9–8.0%) the criteria for PTSD profile by the TES (Table 2). Overall, 8.6% (95% CI, 6.3–11.3%), 4.0% (95% CI, 2.5–6.0) and 2.5% (95% CI, 1.3–4.2%) of women had IES scores ≥20, ≥26, and ≥31, respectively. Moreover, 11.1% of women (95% CI, 8.6–14.0%) had an EPDS score of 12 or greater (Table 2).

**Table 2:** Psychological status of respondents one year after a vaginal delivery (n = 549).

**Depression (**n = 541)		% (95% Confidence Interval) unless otherwise specified
EPDS score (median [IQR])	4	[1–8]
EPDS score ≥12	60/541	11.1 (8.6–14.0)
**Anxiety** (n = 515)		
STAI form Y score (median [IQR])	36	[28–44]
STAI form Y ≥45	127/515	24.7 (21.0–28.6)
**PTSD**		
IES score (median [IQR]) (n = 526)	5	[0–11]
IES ≥20	45/526	8.6 (6.3–11.3)
IES ≥ 26	21/526	4.0 (2.5–6.0)
IES ≥31	13/526	2.5 (1.3–4.2)
**TES**		
Criterion A (trauma)	133/539	24.7 (21.1–28.5)
Criterion B (intrusion)	113/544	20.8 (17.4–24.4)
Criterion C (avoidance/numbing)	65/535	12.1 (9.5–15.2)
Criterion D (persistent arousal)	184/542	33.9 (30.0–38.1)
PTSD profile*	30/527	5.7 (3–9–8.0)
PTSD diagnosis*	22/520	4.2 (2.7–6.3)

EPDS, Edinburgh Postnatal Depression Scale; STAI, State-Trait Anxiety Inventory; PTSD, post-traumatic stress disorder; IES, Impact of Event Scale; TES, Traumatic Event Scale. *The term PTSD diagnosis is used when all diagnostic criteria of the DSM-IV (A, B, C, D, E, F) on the TES scale were met. The term PTSD profile is used when all symptom criteria (B, C and D)^1^ were met, but some other criteria (A, E or F) were missing^4^.

Characteristics significantly associated with a higher risk of PTSD profile by the TES were previous abortion, previous postpartum hemorrhage, hospitalization during pregnancy, instrumental delivery, episiotomy, and postpartum hemoglobin level less than 9 g/dl (Table 3). On multivariate analysis, factors independently associated with PTSD profile were previous abortion (adjusted OR [aOR] 3.6, 95% CI 1.4–9.3), previous postpartum hemorrhage (aOR 5.3, 95% CI 1.3–21.4), and postpartum hemoglobin level less than 9 g/dl (aOR 2.7, 95% CI 1.0–7.5) (Table 4). Characteristics significantly associated with an IES score ≥26 were previous abortion, duration of labor greater than 6 hours, instrumental delivery, and postpartum hemoglobin level less than 9 g/dl (Table 3). On multivariate analysis, factors independently associated with an IES score ≥26 were previous abortion (aOR 4.0, 95% CI 1.3–11.9) and hemoglobin level less than 9 g/dl (aOR 4.0, 95% CI 1.3–11.9) (Table 4).

**Table 3:** Univariate analysis of factors associated with PTSD at one year after vaginal delivery.

**Women’s characteristics**	**PTSD profile by TES scale** ^**1**^ **(n = 527)**				**IES score** ≥**26 (n = 526)**			
	No (n = 497)	Yes (n = 30)	OR	95% CI	No (n = 505)	Yes (n = 21)	OR	95% CI
**Age (years)**								
<25	52 (10.5)	5 (16.7)	1.6	0.6–4.4	55 (10.9)	3 (14.3)	1.4	0.4–5.1
25–34	364 (73.2)	22 (73.3)	ref.	ref.	367 (72.7)	14 (66.7)	ref.	ref.
≥35	81 (16.3)	3 (10.0)	0.6	0.2–2.1	83 (16.4)	4 (19.0)	1.3	0.4–3.9
French nationality	493 (99.1)	28 (96.6)	0.2	0.02–2.1	498 (98.8)	21 (100.0)	—	—
**Tobacco use**								
Never	358 (72.5)	17 (58.6)	ref.	ref.	365 (72.8)	14 (66.7)	ref.	ref.
Never during pregnancy	67 (13.5)	7 (24.1)	2.2	0.9–5.5	67 (13.4)	3 (14.3)	1.2	0.3–4.2
Yes during pregnancy	69 (14.0)	5 (17.2)	1.5	0.5–4.3	69 (13.8)	4 (19.0)	1.5	0.5–4.7
**Body mass index (kg/m** ^**2**^ **)**								
<18.5	32 (6.4)	0 (0.0)	—	—	32 (6.3)	0 (0.0)	—	—
18.5–24.9	365 (73.4)	20 (69.0)	ref.	ref.	364 (72.2)	15 (71.4)	ref.	ref.
25.0–29.9	74 (14.9)	6 (20.7)	1.6	0.6–4.1	77 (15.3)	5 (23.8)	1.7	0.6–4.9
≥30	26 (5.2)	3 (10.3)	2.3	0.6–8.2	31 (6.2)	1 (4.8)	0.8	0.1–6.7
Nulliparous	228 (46.1)	15 (51.7)	1.2	0.6–2.6	233 (46.4)	10 (47.6)	1.0	0.4–2.5
Previous abortion	44 (8.9)	7 (24.1)	3.3	1.3–8.1	45 (9.0)	5 (23.8)	3.2	1.1–9.1
Previous PPH	11 (2.2)	3 (10.3)	5.1	1.3–19.4	13 (2.6)	1 (4.8)	1.9	0.2–15.1
Uterine scar	30 (6.0)	1 (3.4)	0.6	0.1–4.2	30 (5.9)	2 (9.5)	1.7	0.4–7.5
Complication during pregnancy*	92 (18.8)	5 (18.5)	1.0	0.4–2.6	96 (19.4)	1 (5.3)	0.2	0.03–1.7
Hospitalization >24 h	44 (8.9)	6 (20.7)	2.7	1.0–6.9	49 (9.7)	3 (14.3)	1.5	0.4–5.5
**Labor characteristics**								
Induction of labor	75 (15.1)	4 (13.3)	0.9	0.2–2.6	78 (15.4)	6 (28.6)	2.2	0.7–6.2
Duration of labor >6 h	165 (33.3)	13 (44.8)	1.6	0.8–3.5	171 (34.0)	12 (57.1)	2.6	1.1–6.2
Epidural analgesia	477 (96.0)	28 (97.0)	1.2	0.1–9.1	483 (95.8)	21 (100.0)	—	—
Oxytocin during labor**	390 (78.5)	25 (86.2)	1.7	0.6–5.0	399 (79.2)	19 (90.5)	2.5	0.6–10.1
Instrumental delivery	55 (11.1)	7 (23.3)	2.4	1.0–6.0	59 (11.7)	7 (33.3)	3.8	1.5–9.7
Episiotomy	190 (38.2)	17 (56.7)	2.1	1.0–4.4	201 (39.8)	11 (52.4)	1.7	0.7–4.0
3^rd^- or 4th-degree perineal lacerations	6 (1.2)	1 (3.3)	2.8	0.3–24.2	6 (1.9)	1 (4.8)	4.2	0.5–36.2
Manual removal of placenta	30 (6.0)	2 (6.7)	1.1	0.2–4.9	29 (5.7)	1 (4.8)	0.8	0.1–6.3
Blood loss ≥500 ml	54 (10.9)	4 (13.3)	1.3	0.4–3.7	56 (11.1)	3 (14.3)	1.3	0.4–4.7
Blood loss ≥1,000 ml	12 (2.4)	1 (3.3)	1.4	0.2–11.1	12 (2.4)	2 (9.5)	4.3	0.9–20.6
**Immediate postpartum period**								
Hemoglobin level <9 g/dl at day 2	42 (8.5)	6 (21.4)	2.9	1.1–7.6	45 (9.0)	5 (26.3)	3.6	1.2–10.4
**Neonatal outcome**								
Birth weight (g)								
<2500	6 (1.2)	1 (3.4)	3.0	0.3–26.3	6 (1.2)	1 (4.8)	4.5	0.5–40.0
2500–3999	457 (92.0)	25 (86.2)	ref.	ref.	464 (92.1)	17 (80.9)	ref.	ref.
>4000	34 (6.8)	3 (10.3)	1.6	0.5–5.6	34 (6.7)	3 (14.3)	2.4	0.7–8.6
5-min Apgar score <7	1 (0.2)	0 (0.0)	—	—	1 (0.2)	0 (0.0)	—	—
pH <7.00	0 (0.0)	0 (0.0)	—	—	0 (0.0)	0 (0.0)	—	—
Transfer to neonatal intensive care unit	13 (2.6)	1 (3.4)	1.3	0.2–10.5	12 (2.4)	2 (9.5)	4.3	0.9–20.5

Data are no. (%) unless otherwise specified. PTSD, post-traumatic stress disorder; IES, Impact of Event Scale; TES, Traumatic Event Scale. ^1^Criteria B, C and D on TES. *Complication during pregnancy including thrombocytopenia, gestational diabetes, bleeding, hypertensive disorders, or preterm labor. **First and second stage.PPH, postpartum hemorrhage.

**Table 4:** Multivariate analysis of factors associated with PTSD at one year after vaginal delivery.

	**PTSD profile by TES scale** ^**1**^				**IES score** ≥**26**			
	**Model 1**		**Model 2**		**Model 1**		**Model 2**	
	**aOR**	**95% CI**	**aOR**	**95% CI**	**aOR**	**95% CI**	**aOR**	**95% CI**
**Previous abortion**	3.1	1.2–7.8	3.6	1.4–9.3	3.1	1.0–9.0	4.0	1.3–11.9
**Previous PPH**	5.8	1.4–23.7	5.3	1.3–21.4	NS in univariate analysis		NS in univariate analysis	
**Hospitalization during pregnancy**	2.4	0.9–6.4	1.8	0.6–5.2	NS in univariate analysis		NS in univariate analysis	
**Labor >6 h**	NS in univariate analysis		NS in univariate analysis		2.1	0.8–5.3	Not included	
**Instrumental delivery**	2.0	0.7–5.7	Not included		2.7	0.98–7.5	Not included	
**Episiotomy**	1.6	0.6–3.8	Not included		NS in univariate analysis		NS in univariate analysis	
**Hb postpartum** <**9 g/dl at day 2**	Not included		2.7	1.0–7.5	Not included		4.0	1.3–11.9

For each model, variables included are those listed. aOR, adjusted odds ratio; CI, confidence interval; PTSD, post-traumatic stress disorder; IES, Impact of Event Scale; TES, Traumatic Event Scale. ^1^Criteria B, C and D on TES.

Among the 56 respondents (10.3%) who reported bad memories of childbirth at day 2, 21.1% (11/52) had a PTSD diagnosis at one year and 21.1% (11/52) a PTSD profile, compared with 2.4% (11/467; OR 11.1, 95% CI 4.1–30, *P* < 0.001) and 3.8% (18/467; OR 6.7, 95% CI 2.6–16.1, *P* < 0.001), respectively, of the 489 (87.3%) women who did not report such feelings at day 2 (Table 4). The proportion of women with one-year IES scores of 20, 26, and 31 or greater was 31.5%, 21.4%, and 16.7%, respectively, among women who reported bad memories at day 2, compared to 5.8% (*P* < 0.001), 2.1% (p < 0.001) and 0.9% (*P* < 0.001), respectively, for women not reporting a bad memory (Table 5). The EPDS and STAI scores at one year postpartum did not differ by presence of bad memories of childbirth at day 2 post partum.

**Table 5:** Association between bad memories of birth in immediate postpartum period (day 2) and psychological status at one year.

	Good memories of birth at day 2 n = 489		Bad memories of birth at day 2 n = 56		*P* value
**Depression**	n	%	n	%	
EPDS score (median [IQR])	479	4 [1–7]	56	4 [1–8.5]	0.95
EPDS score ≥12	50	10.4	8	14.3	0.38
**Anxiety**					
STAI form Y score (median [IQR])	457	35 [28–44]	52	37.5 [29–48]	0.16
STAI form Y score ≥45	106	23.2	18	34.6	0.07
**PTSD**					
IES score (median [IQR])	466	5[0–11]	54	7 [3–22]	0.004
IES ≥20	27	5.8	17	31.5	<0.001
IES ≥26	10	2.1	11	21.4	<0.001
IES ≥31	4	0.9	9	16.7	<0.001
**TES**					
Criterion A (trauma)	98	20.5	33	58.9	<0.001
Criterion B (intrusion)	91	18.9	20	35.7	0.003
Criterion C (avoidance/numbing)	50	10.5	13	24.5	0.003
Criterion D (persistent arousal)	158	32.8	22	40.7	0.24
PTSD profile*	18	3.8	11	21.1	<0.001
PTSD diagnosis*	11	2.4	11	21.1	<0.001

EPDS, Edinburgh Postnatal Depression Scale; STAI, State-Trait Anxiety Inventory; PTSD, post-traumatic stress disorder; IES, Impact of Event Scale; TES, Traumatic Event Scale. *The term PTSD diagnosis is used when all diagnostic criteria of the DSM-IV (A, B, C, D, E, F) on the TES scale were met. The term PTSD profile is used when all symptom criteria (B, C and D)^1^ were met, but some other criteria (A, E or F) were missing^4^.

## Discussion

Our results suggest that one year after a vaginal delivery, about 1 in 20 women has a PTSD profile and 1 in 40 meets the criteria for PTSD diagnosis. PTSD at one year was markedly more prevalent in women who reported bad memories of childbirth at day 2 post partum. Previous abortion and postpartum anemia were significant risk factors for PTSD according to both the TES and IES.

### Comparison with other studies

To our knowledge, our study is the first to prospectively assess PTSD in a low-risk population, one year after vaginal delivery. The available literature provides sparse and conflicting results regarding the course of PTSD after childbirth, and whether its prevalence increases, decreases, or remains unchanged over time, remains unclear^30^. Our results could not resolve this issue, but they demonstrate that the prevalence of PTSD diagnosis and profile remains significant at one year after a vaginal delivery.

Although we were able to study many characteristics of pregnancy, labor, delivery, and the immediate postpartum period, only a low hemoglobin level at day 2 post partum was a significant risk factor for a PTSD profile. In particular, consistent with previous reports^2, 4, 30^, we found no association between episiotomy and PTSD. Nonetheless, multiple regression analysis in some studies has revealed instrumental delivery as a risk factor of PTSD, but at 4–6 weeks^31^ and 2–6 months^18^ after delivery. Future prospective studies are required to determine the impact of instrumental delivery and episiotomy at one year after delivery. We found these two variables were associated with PTSD on univariate analysis in our study; the association became non-significant on multivariate analysis, although this may be due to lack of power. Moreover, similar to previous reports^18, 32^, we did not find postpartum hemorrhage associated with subsequent PTSD. Some authors have found that severe postpartum hemorrhage may have a long-term psychological impact on women despite uterine preservation^33^. However, our study was probably under-powered to explore this rare outcome, because the rate of blood loss of 1,000 mL or greater was only 2.5% in our sample.

### Implications for clinical practice

Interestingly, we found that a hemoglobin level less than 9 g/dl at day 2 post partum was an independent risk factor for a PTSD profile by the TES as well as for a high IES score. This suggests that excessive peripartum blood loss may affect women’s psychological status and play a role in the occurrence of PTSD after delivery. This result is consistent with the literature because postpartum anemia has been associated with mood and depressive disorders^34^ with a negative correlation between hemoglobin level and depressive symptoms^35^. Interestingly, iron deficiency, a common cause of anemia, alters thyroid hormone metabolism^35^, while the hypothalamic-pituitary-thyroid axis involved in stress-related syndromes is suspected to play a role in PTSD because it is known that trauma can trigger thyroid abnormalities^36^. Our results strongly support the need for health-care providers to try to prevent iron deficiency during the postpartum period, as recommended by several expert groups^37–39^. Finally, we report an association between previous abortion and PTSD following childbirth assessed by the TES or by an IES score of 26 or greater. This result is consistent with the literature reporting long-term anxiety and depression following abortion^40^.

Detecting women at high risk of PTSD after delivery is a current focus in research and clinical obstetrics. For pragmatic reasons, including the need to screen a large number of women to detect the small proportion of women at risk, the use of a routine PTSD interview or even self-administered questionnaire seems impractical. In our study, we found that the answer to a simple question, “Today, what are your memories of your childbirth?” asked during the postpartum period was strongly associated with PTSD profile or diagnosis at one year after delivery. Accordingly, this question might be a quick and simple tool that could be used routinely to identify women at high risk of PTSD; this preliminary result should however be confirmed by other prospective studies in other populations. We can even hypothesize that early intervention in the immediate postpartum period may reduce PTSD symptoms, as it has been shown to decrease post-traumatic stress reactions in other trauma contexts or exposures^41, 42^, but this must be tested in this specific context.

### Strengths and weaknesses of the study

The main strengths of our study are its prospective design, with a large cohort and long-term follow-up at one year after delivery, the availability of detailed data regarding pregnancy, labor, delivery, and the immediate postpartum period, in particular, peripartum blood loss and related outcomes such as postpartum hemoglobin level, as well as about the woman’s feelings about her delivery at day 2. The quality of these data, extracted from the database of an RCT related to prevention of postpartum hemorrhage, was checked^15^. Moreover, the restriction of inclusion to women at low obstetric risk (i.e., late preterm and term singleton vaginal deliveries) eliminates potential confounding biases that may interfere with PTSD, such as emergency cesarean section^43^ or moderate or severe preterm delivery^30, 44^.

Our study also has some limitations. First, the definition of PTSD was based on DSM-IV criteria, because DSM-V, which changed this definition substantially, was published after our study began^45^. However, TES was developed specifically for PTSD after childbirth^16–19^, and allows comparisons of our results with the existing literature. Moreover, we also used IES, because it is one of the most frequently self-reported scales and provides a good indicator of PTSD^4^. Second, the response rate was only 49.8%, which was nonetheless similar to that of other studies involving mailed self-reported scales to assess PTSD in the postpartum period at a shorter interval after delivery^4^. Third, respondents were at lower obstetric risk than non-respondents, so that the prevalence of PTSD in our sample may actually underestimate the rate in the general low-risk population. Moreover, the most robust way to assess PTSD is to use clinical interviews by a trained clinician to limit the possibility of measurement errors of PTSD diagnosis and profile^4, 30^. The feasibility and cost of such clinical interviews, however, is prohibitive in view of the low prevalence of PTSD after childbirth, which explains why all studies assessing PTSD post partum have used self-reported scales^4, 30^. However, our results (prevalence, risk factors, and association with bad memories at day 2) for women with a PTSD profile were quite similar whether assessed by the TES or by an IES score of 26 or greater. This finding suggests that the risk of measurement errors of PTSD profile is low.

Fourth, although our data about pregnancy, labor, delivery, and the immediate postpartum period were robust and useful for identifying risk factors of PTSD, relevant confounding factors such as history of depression, sexual abuse, and trait anxiety^2^ were unfortunately unavailable because the this study was designed during the TRACOR trial. In addition, although our sample is one of the largest published^4^, its size does not provide sufficient power for the adequate exploration of risk factors with low prevalence. Finally, although the TES is designed to focus on childbirth, we cannot rule out by possibility that another traumatic life event may have occurred since delivery and contributed to the emergence of a PTSD profile. The scales we used do not delineate clearly between current PTSD, caused by childbirth, and pre-existing/lifetime PTSD, caused by another incident trauma, that becomes symptomatic following childbirth. This point does not, however, impair the validity or detract from the implications of our results for the health-care providers in their daily clinical practice.

In conclusion, PTSD following childbirth –whether related to or associated with it- is not rare a year after vaginal delivery in a low-risk population and is more frequent in women with a history of previous abortion and a hemoglobin level less than 9 g/dl at day 2 post partum. Bad memories of deliveries at day 2 reported by women answering this one simple question were strongly associated with PTSD diagnosis and profile a year after vaginal delivery. This question might be a useful tool that could be routinely used to detect women at high risk of PTSD.
